# A case-report of the unprovoked thrombotic event in a patient with thymoma and severe FVII deficiency

**DOI:** 10.1186/s12959-023-00494-3

**Published:** 2023-05-04

**Authors:** Lei Li, Xi Wu, Wenman Wu, Qiulan Ding, Xuefeng Wang

**Affiliations:** 1 Department of Laboratory Medicine, Ruijin Hospital, Shanghai Jiao Tong University School of Medicine, Shanghai, China; 2 State Key Laboratory of Medical Genomics, Shanghai Institute of Hematology, Ruijin Hospital, Shanghai Jiao Tong University School of Medicine, Shanghai, China; 3 Collaborative Innovation Center of Hematology, Shanghai Jiao Tong University School of Medicine, Shanghai, China

**Keywords:** Factor VII deficiency, Thymoma, Venous thromboembolism, Protein S, Thrombin generation assay

## Abstract

**Background:**

Factor VII deficiency is a rare bleeding disorder caused by a deficiency of clotting factor VII. However, there have been some case reports of venous thrombosis in patients with factor VII deficiency, especially underlying the prothrombotic risk factors exposure. Patients with factor VII deficiency require special considerations before undergoing surgery to minimize the risk of bleeding or thrombogenesis.

**Case presentation:**

Here, we described a patient with early-stage thymoma and severe factor VII deficiency who experienced an unprovoked thrombotic episode before thymectomy and a fatal thrombotic event after surgery. By adopting gene screening, a reported homozygous *F7* mutation (p.His408Gln) and a novel heterozygous *PROS1* mutation (p.Pro147Ala) were identified. The former resulted in severe factor VII deficiency but did not protect against thrombosis, and the latter was correlated with normal expression and cofactor activities of protein S through the thrombin generation test. The perioperative infusion of recombinant factor VII concentrate and the absence of antithrombotic prophylaxis may collectively contribute to her fatal thrombotic event after surgery.

**Conclusions:**

For the patients with severe factor VII deficiency undergoing surgery, uniform replacement therapy may not be recommended, and antithrombotic prophylaxis should be used in the case with thrombotic history to minimize the risk of bleeding and thrombogenesis.

## Background

Venous thromboembolism (VTE), mainly including deep vein thrombosis and pulmonary embolism, represents a significant healthcare burden worldwide. With increased recognition from both patients and physicians, combined with comprehensive strategies and accurate diagnostic methods, more and more VTE patients can be identified and treated promptly [1]. The most widely assessed genetic factors associated with VTE include loss of function in anticoagulant factors (antithrombin, protein C, and protein S (PS)), or gain of function in procoagulants such as factor V Leiden and prothrombin G20210A [2]. Paradoxically, many patients with either venous or arterial thrombotic events were identified with inherited bleeding disorders, with afibrinogenemia, factor XI deficiency, and factor VII (FVII) deficiency being the most commonly associated with thrombosis [3, 4]. Previous reviews supported that the occurrence of VTE, especially deep vein thrombosis, has been reported in 3–4% of patients with FVII deficiency, even in severely deficient patients [5–7]. Most patients with FVII deficiency who developed thrombosis were associated with known prothrombotic risk factors [8], such as advanced age, surgery, replacement therapy, et al.

In this study, we described a female patient with early-stage thymoma and severe FVII deficiency who experienced an unprovoked thrombotic episode before thymectomy and a fatal thrombotic event after surgery. Her clinical features, laboratory characteristics, and genetic background were summarized. This case report demonstrated that, even for patients with severe factor VII deficiency, the application of replacement therapy or anticoagulant treatments should be carefully determined based on their thrombotic history to minimize the risk of bleeding and thrombogenesis.

## Case presentation

This study was approved by the ethics committee of Ruijin Hospital and informed consent was obtained from all participants. The patient, a 53-year-old Chinese woman who was suspected of having a thymic tumor through a routine physical examination one year ago, was admitted to a local hospital for thymectomy. The thymic tumor of this patient was clinically diagnosed as stage II thymoma (Masaoka system) [9] by imaging examinations without invasion of surrounding structures. During preoperative testing, thrombi were surprisingly observed in her left lower lobe of the lung and right lower extremity, but this patient had no subjective symptoms. No acquired factors which contributed to thrombotic risk including old age (age over 60 years), hormone replacement therapy, oral contraceptives, malignancy, obesity, smoking, dehydration, diabetes, hyperhomocysteinemia, polycythemia, varicose veins, COVID-19 infection, et al., were observed. The results of haemostasis analysis showed an elevated D-dimer (2.68 µg/mL) and prolonged prothrombin time (PT, 32.1s). Measurements of coagulation factors indicated that she had severe FVII deficiency (FVII activity (FVII:A), 2.4%). The patient denied any history of anemia or spontaneous bleeding, including epistaxis, hemoptysis, or hematuria. The family history of thrombosis and bleeding were negative.

After two-month anticoagulation therapy, the patient was eligible for thoracoscopic thymectomy. A 20 µg/kg dose intravenous infusion of recombinant activated FVII (rFVIIa) was given before starting surgery. Surgical biopsy and histological analysis demonstrated a type AB (World Health Organization Classification system) thymoma of stage I (TNM system) [10, 11]. After surgery, rFVIIa was repeatedly administered (15 µg/kg) every 8 h for the first 24 h. Two days after the operation, she suddenly developed acute respiratory failure and was given tracheal intubation and assisted ventilation. Multiple embolisms in bilateral pulmonary arteries and left lower extremity were observed through imaging examinations. Low molecular weight heparin and norepinephrine pump were then applied. However, followed by a progressive decrease of platelet (219 g/L, 93 g/L, and 75 g/L, reference interval 100–300 g/L), low molecular weight heparin was discontinued. Inferior vena cava filter implantation and pulmonary arteriography and dilatation were performed. In the postoperative treatments of anti-infection, fluid replacement, and rivaroxaban anticoagulant, the patient gradually recovered.

Three months later, the patient was referred to our center by local clinicians for screening the thrombotic risk factors. Two weeks after ceasing rivaroxaban treatment, haemostatic tests including clotting function tests, activities of anticoagulant proteins, and plasma levels of coagulation factors were performed as previously described in the patient and related family members (Table 1) [12, 13]. Patient had prolonged PT and severely reduced FVII:A, while other coagulation factors and anticoagulant proteins were normal. Antiphospholipid syndrome is excluded by repeatedly negative autoimmune antibodies (lupus anticoagulant, anticardiolipin antibody, and anti-β2 glycoprotein antibody 1).

**Table 1:** Laboratory characteristics of the patient and relative family members in this study

	PT (s)		APTT (s)	FDP (mg/L)	DD (mg/L)	AT:A (%)	PC:A (%)	PS:A (%)	FPS:Ag (%)	TPS:Ag (%)	LAC	aCL (PL/ml)	b2-GPI (RU/ml)	FVII:A (%)	FVII:Ag (%)	FVIII:A (%)	FIX:A (%)	FX:A (%)	vWF:A (%)
Patient		28.1	32.9	1.5	0.35	102	104	86.3	99	111	1.1	3	5	2.6	3.4	163.4	149.9	71	158
Mother		15.0	30.8	1.3	0.23	114	110	94	100	120	1.1	5	7	63	58	145	149	80	143
Son		14.4	36.8	1.2	0.13	98	100	82	89	104	1.0	6	3	57	59	120	132	70	140
Daughter		13.1	28.7	2.0	0.21	96	98	86	94	121	1.0	3	3	68	62	126	112	68	136
Ref.		10 | 16	22.3 | 38.7	< 5	< 0.55	85 | 120	70 | 140	60 | 130	60 | 130	60 | 130	< 1.2	< 12	< 12	50 | 150	50 | 150	50 | 150	50 | 150	50 | 150	60 | 150

PT, prothrombin time; APTT, activated partial thromboplastin time; FDP, fibrinogen degradation products; DD, D-dimer; AT, antithrombin; PC, protein C; PS, protein S; FPS, free protein S; TPS, total protein S; LAC, lupus anticoagulant; aCL, anticardiolipin antibody;β2-GPI, anti-β2 glycoprotein I; FVII, factor VII; FVII, factor VIII; FIX, factor IX; FX, factor X; vWF, von willebrand factor; A, activity; Ag, antigen; Ref., normal ranges for corresponding laboratory characteristics

**Fig. 1:**
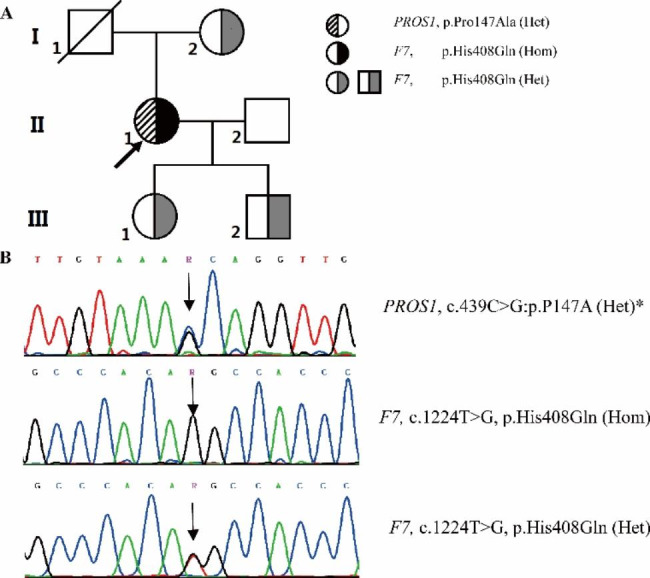
Family pedigrees, genetic sequencing of *F7* p.His408Gln and *PROS1* p.Pro147Ala mutation. A. The family pedigree. Arrow, patients; slashed symbol, family member passed away; Circle, female; square, male. B, Chromatogram of DNA sequence covering the flank region of mutation (c.439 C > G:p.Pro147Ala) in exon 5 of *PROS1* and mutation (c.1224T > G, p.His408Gln) in exon 9 of *F7*. The numbering of changed amino acid residue was numbered by regarding the start codon as residue 1. Het, heterozygote; Hom, homozygote; *, novel mutation

Then, genetic analysis including 35 genes related to VTE was carried out using two high throughput next generation sequence and copy number variation detection. To remove polymorphisms, novel mutations were filtered against the database from 1000 Genomes Project (http://www.1000genomes.org/). The bioinformatics including PolyPhen-2, SIFT, MutationTaster, and Align GVGD were used to remove benign variations as previous report [13]. Through gene screening, a reported homozygous *F7* mutation (c.1224T > G, p.His408Gln) and a novel heterozygous *PROS1* gene mutation (c.439 C > G:p.Pro147Ala) were identified (Fig. 1). In the current case, homozygous His408 to Gln mutation in *F7* resulted in severe FVII deficiency (FVII:A 2.6%, FVII:Ag: 3.4%, 50–150%). Heterozygous p.His408Gln in *F7* was identified in the patient’s mother, daughter, and son, who had FVII levels between 40 and 60%. Additionally, the patient’s father was unavailable for testing because he had passed away. The patient denied any history of consanguinity. None of above investigated family members presented any bleeding or thrombosis symptoms.

**Fig. 2:**
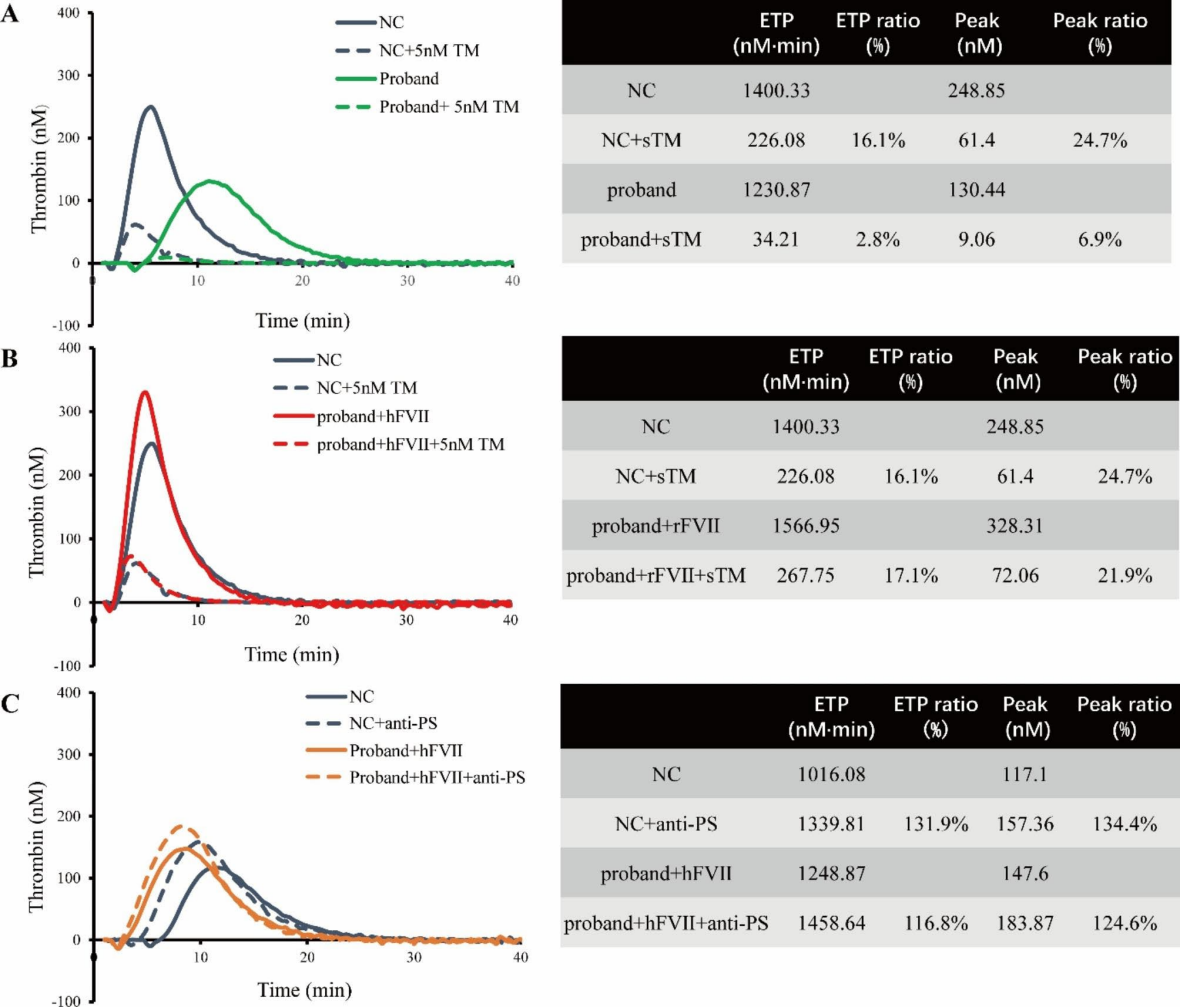
Thrombin generation test in plasma from normal control and patient. (A) APC/PS cofactor function detected by thrombin generation test using control PPP and patient PPP without supplementation of hFVII; (B) APC/PS cofactor function detected by thrombin generation test using control PPP and patient PPP supplemented with hFVII; (C) TFPI/PS cofactor function detected by thrombin generation test using control PPP and patient PPP supplemented with hFVII. ETP, endogenous thrombin potential; NC, normal control; PPP, pool platelets plasma; rFVIIa, recombinant activated factor VII; sTM, soluble recombinant human thrombomodulin

*PROS1* p.Pro147Ala mutation was first reported in this study. Except for proband, it has not been detected in any other family members (Fig. 1). Two of the bioinformatic predictions (MutationTaster and Align GVGD) showed probably damaging results, while the other two predictions (PolyPhen-2 and SIFT) showed tolerated results. Patient with *PROS1* p.Pro147Ala mutation had normal levels of PS antigen and activity. However, PS activity here referred to the property of PS to act as a cofactor to activated protein C (APC) but not a cofactor to tissue factor pathway inhibitor (TFPI). Therefore, thrombin generation test (TGT) was used to evaluate the APC and TFPI cofactor activity of PS in this patient [14]. Due to the decreased FVII, the patient’s pool platelets plasma (PPP) showed an obviously hypocoagulable state compared with normal control, which strongly interfered the measurement by TGT (Fig. 2 A). Therefore, the patient’s PPP was added with plasma-derived human FVII (hFVII) to obtain a physiological concentration of FVII (500ng/ml) for the following experiments. Firstly, to determine the APC cofactor activity of PS, TGT was initiated with PPP 5 pM reagent adding in PPP-hFVII or PPP-control in the absence and presence of 5 nM soluble recombinant human thrombomodulin (sTM). The APC cofactor activity of PS was expressed as the ratio of the endogenous thrombin potentials obtained with and without sTM. As shown in Fig. 2B, the APC-cofactor activity of PS between the patient (17.1%) and normal control (16.1%) were comparable. Secondly, to determine the TFPI cofactor activity of PS, TGT was initiated with PPP 1 pM reagent [15]. PPP-hFVII or PPP-control was incubated at 37 °C with 10 µL Hepes-NaCl buffer in the absence or presence of 2.80 µM (final concentration) polyclonal antibodies against PS (anti-PS) for 15 min. The TFPI cofactor activity of PS was expressed as the ratio of the peaks obtained in the absence and presence of anti-PS. From Fig. 2 C, we noticed a comparable TFPI-cofactor activity of PS in the patient (124.6%) and normal control (134.4%). Therefore, the novel PS variant (p.Pro147Ala) did not impair APC and TFPI cofactor activities of PS.

## Discussion

In our previous study, we systematically described the mutation spectrum of *F7* gene in 50 unrelated Chinese patients with FVII deficiency, and we revealed that p.His408Gln was constituted a hotspot mutation of *F7* gene in Chinese population with an incidence of 38.0% (19/50) [16]. About 14% (7/50) patients were identified with homozygous p.His408Gln in the absence of consanguine marriage, whose clinical manifestations were quite variable in different individuals [16]. The current case demonstrated that low FVII induced from homozygous p.His408Gln did not protect from developing VTE.

As reported, 3 to 4% of patients with FVII deficiency have VTE episodes whereas arterial thrombosis is rare, but the sporadic thrombotic events occurring in FVII deficiency patients without any associated prothrombotic factors are rare [4, 5]. In a review of 33 patients with FVII deficiency coexisted with arterial or venous thrombosis, 30 of them carried at least one acquired prothrombotic risk factor (surgery, old age, and replacement therapy) [8]. In the current study, the patient was first diagnosed with VTE during preoperative examinations, while no other acquired risk factors for VTE were identified other than thymoma, which was diagnosed as stage II (Masaoka system) disease preoperatively and stage I (TNM system) postoperatively with no invasion of surrounding structures. To date, rare evidence had supported that patients with early-stage thymoma had a higher risk to develop VTE [17–19]. Distinguishing between provoked and unprovoked VTE is critical for the management of VTE, because patients with unprovoked VTE have a significantly increased risk of recurrence, as compared with patients who have provoked VTE [20]. For the current patient, lifelong or indefinite anticoagulant treatment may be indicated, especially when she is exposed to strong, transient provoking factors, such as major surgery, trauma, or immobility.

In the current study, we reviewed her treatment process and found some inappropriate operations. On the one hand, due to the use of rFVIIa has been linked to thrombotic events in congenital bleeding disorders, especially in FVII deficiency, replacement therapy must be individualized based on the history of hemostatic challenges, family history of bleeding and thrombosis, as well as the level of FVII (more than 20%, or not) and the type of surgery [4, 21]. In the current case, the intraoperative infusion of rFVIIa may partially contribute to the thrombotic event after surgery. On the other hand, antithrombotic prophylaxis must be individualized according to the history of thrombosis and the level of FVII. However, safety, treatment modalities, and specific indications of such antithrombotic prophylaxis remain to be established [22]. Prof. Schved recommended that antithrombotic prophylaxis must be considered for patients with FVII levels greater than 30%, or between 10 and 30% in cases of thrombosis history, but for the severe cases (FVII activity < 10%), pharmacological postoperative thromboprophylaxis is not systematically performed concerning surgical situations in the absence of therapeutic recommendations [22]. In the current case, the absence of antithrombotic prophylaxis may also partially lead to the fatal thrombotic episode after surgery. Thus, we considered that antithrombotic prophylaxis should be used for patients with a thrombosis history, even if the patients had severe FVII deficiency.

The heterozygous *PROS1* mutation (p.Pro147Ala) correlated with normal levels of FPS:Ag and PS:A was first reported in this study. PS is a multifunctional anticoagulant that not only enhances the inactivation of activated factors V and factor VIII as the cofactor of APC but also directly inhibits free activated factor X as a cofactor of full-length TFPI [13, 23]. The two cofactor functions of PS may be independent. Thus, type II PS-deficient patients may have mutations that selectively impair one of the two cofactor functions of PS and hence may have differential effects on the APC- and TFPI-cofactor activities. In general, clinicians diagnosed PS deficiency according to the PS level in plasma measured with functional and immunological assays. However, most functional PS assays are based on measuring the ability of PS to prolong the clotting time of plasma as a cofactor to APC rather than TFPI. Thus, *PROS1* mutations that selectively impair TFPI cofactor functions of PS may be missed diagnosis. In recent years, a method based on the measurement of thrombin generation in the absence and presence of neutralizing antibodies against PS has been used to analyze the TFPI-cofactor activity of PS [13, 24]. In the current study, though the novel *PROS1* variant (p.Pro147Ala) did not show impaired TFPI cofactor activity, it is still an essential detection to improve the diagnosis of PS deficiency.

## Conclusion

To conclude, we described a patient with thymoma and severe FVII deficiency who experienced an unprovoked thrombotic episode before thymectomy and a fatal thrombotic event after surgery. A homozygous *F7* mutation (p.His408Gln) and a heterozygous *PROS1* variant (p.Pro147Ala) were identified. The former resulted in severe FVII deficiency that did not protect against thrombosis, and the latter was correlated with normal expression and cofactor functions of PS. The intraoperative infusion of rFVIIa and the absence of antithrombotic prophylaxis may collectively contribute to her fatal thrombotic event after surgery. Thus, for the patients with severe factor VII deficiency undergoing surgery, uniform replacement therapy may not be recommended, and antithrombotic prophylaxis should be used in the case with thrombotic history to minimize the risk of bleeding and thrombogenesis.

## Data Availability

The datasets used and/or analyzed during the current study are available from the corresponding author on reasonable request.

## Abbreviations

APC: activated protein C
FPS: Ag: free protein S antigen
FVII: factor VII
FVII: A: factor VII activity
hFVII: plasma-derived human factor VII
PT: prothrombin time
PPP: pool platelets plasma
PS: protein S
rFVIIa: recombinant activated factor VII
sTM: soluble recombinant human thrombomodulin
TFPI: tissue factor pathway inhibitor
TGT: thrombin generation test
TPS: Ag: total protein S antigen
VTE: venous thromboembolism
